# The opioid prescribing practices of surgeons: A comprehensive review of the 2015 claims to Medicare Part D

**DOI:** 10.1016/j.sopen.2019.05.008

**Published:** 2019-09-11

**Authors:** Syed I. Khalid, Ryan Kelly, Ridha Khalid, Rita Wu, Amilia Y. Ni, Owoicho Adogwa, Joseph Cheng

**Affiliations:** a Department of Surgery, Rush University Medical Center, Chicago, IL; b Georgetown University School of Medicine, Washington, DC; c Department of Neurological Surgery, University of Cincinnati Medical Center, Cincinnati, OH; d Department of Neurosurgery, Rush University Medical Center, Chicago, IL; e Chicago Medical School, North Chicago, IL

## Abstract

**Background:**

The Centers for Disease Control and Prevention have declared that the United States is amidst a continuing opioid epidemic, with drug overdose–related death tripling between 1999 and 2014. Among the 47,055 overdose-related deaths that occurred in 2014, 28,647 (60.9%) of them involved an opioid.

**Methods:**

To determine if there are specific trends in opioid prescribing practices of specific groups of surgeons to better describe any regional or subspecialty trends that exist, the Part D Prescriber Public Use File was used to evaluate all prescription drug orders for Medicare beneficiaries with a Part D prescription drug plan for the 2015 calendar year. Only those providers with the specialty description corresponding to a surgical specialty were included in this study, using the provider's Part B claims.

**Results:**

A total of 65,277,932 claims made to Part D by 90,253 surgeons in the 2015 service year were analyzed in this study, demonstrating statistically significant differences in the opioid prescribing practices of surgeons from different states, cities, practice settings, and subspecialties (*P* < .05). During this year, these surgeons' opioid medication claims cost the health care system $133,091,997.81 in drug benefits.

**Conclusion:**

All health professionals with opiate prescribing privileges are entrusted with and responsible for the use of these medications; therefore, physicians have a crucial role in ensuring safe and effective use of this treatment option and the deterrence of its abuse. This is true in particular for surgeons given the acuity level and context of their practice.

## Highlights

• A total of 47,055 overdose deaths occurred in 2014; 28,647 (60.9%) of them involved an opioid.
• Medicare Part D was queried for information on prescription drug events.
• A total of 65,277,932 claims by 90,253 surgeons in the 2015 service year were analyzed.
• Prescribing practices differed from surgeons based on states and subspecialties.
• Opioid medication claims cost the health care system $133,091,997.81 in benefits.

## INTRODUCTION

The Centers for Disease Control and Prevention (CDC) have declared that the United States is amidst a continuing opioid epidemic, with drug overdose–related death tripling between 1999 and 2014 [1], [2]. During this same period, sales of prescription opioids in the United States have nearly quadrupled, with patients older than 40 years more likely to be prescribed opioids as well as more likely to use opioids than younger adults (ages of 20 and 39 years) [5]. Prescribing rates among surgeons (37% of all opioids prescribed) are second only to the prescription rates of pain medicine providers [6]. Surgeons' high rate of opioid prescription is likely motivated by the acuity of their pain treatment and the strong evidence that demonstrates the efficacy of opioid combinations in the treatment of postoperative pain [7], [8], [9].To this end, we have aggregated and analyzed provider-level Part D data from the Centers for Medicare and Medicaid Services (CMS) and the CDC with the goal of understanding the individual opioid prescribing practices of different surgical subspecialties; different practice environments; and cities, states, and regions to better describe potential foci that may help inform surgeons, policy makers, and the health care stake holders at large.

## METHODS

There were 44.1 million people enrolled in Medicare Part D in the 2015 calendar year. This includes those on Medicare who 65 years and older and those on Medicaid who are younger than 65 years with permanent disabilities and have access to the Part D drug benefits. The Part D Prescriber Public Use File (PUF) is based on all beneficiaries enrolled in the Medicare Part D prescription drug program, which includes approximately 70% of all Medicare beneficiaries, an estimated 41.8 million persons. The PUF was used to query data on prescription drug events (PDEs) incurred by all Medicare beneficiaries with a Part D prescription drug plan for the 2015 calendar year. These data included all PDEs received by CMS from June 31, 2015, to June 30, 2016. Only providers with a specialty description considered to be a surgical specialty by The American College of Surgeons, as reported on the provider's Part B claims, were included in this study. These specialties included cardiothoracic surgery, colon and rectal surgery, general surgery, gynecology and obstetrics, gynecologic oncology, neurologic surgery, ophthalmic surgery, oral and maxillofacial surgery, orthopedic surgery, otorhinolaryngology, pediatric surgery, plastic and maxillofacial surgery, urology, and vascular surgery. The National Provider Identifier number of providers was organized by surgical specialty, city, and state. These data were reviewed for Total Claim Count, Total Beneficiary Count, Opioid Beneficiary Count, Opioid Claim Count, Total Day Supply, Opioid Day Supply, Total Drug Cost, and Opioid Drug Cost.

### Definition of an *Opioid Drug*

The list of drugs included in data analysis for an Opioid Drug referred to the Prescriber Drug Category List for opioids, which is based upon drugs in the Medicare Part D Overutilization Monitoring System. This list is originally published by the CDC (https://www.cms.gov/Medicare/Prescription-Drug-Coverage/PrescriptionDrugCovContra/RxUtilization.html).

### Definition of Urbanized Cities

The Census Bureau's urban-rural classification was used to define urban and rural areas. To qualify as an urban area, the territory identified according to criteria must encompass at least 2,500 people, at least 1,500 of which reside outside institutional group quarters. The Census Bureau identifies 2 types of urban areas: Urbanized Areas (UAs) of 50,000 or more people and Urban Clusters (UCs) of at least 2,500 and less than 50,000 people. Both UAs and UCs were counted as urban cities. Rural cities encompassed all population, housing, and territory not included within an urban area.

### Death Rates Adjusted for Age

Deaths were classified using the *International Classification of Diseases, Tenth Revision*. Deaths by drug poisoning were identified using associated cause-of-death codes X40–X44, X60–X64, X85, and Y10–Y14. Death rates adjusted for age were calculated as the number of deaths per 100,000 persons using the direct method and the 2000 standard population. Death by drug overdose data were obtained from the National Center for Injury Prevention and Control, Division of Unintentional Injury Prevention's public database, a branch of the CDC. All calculated data were in accordance with the CDC's calendar year data for 2015.

### Data Analysis

All analyses were performed to reflect the entire cohort. All statistical analyses, paired *t* test, and univariate analysis of variance were performed using R Statistical Software (Version 3.1.1, 2013; Vienna, Austria).

## RESULTS

### Provider Subspecialty Prescribing Practices

**Fig. 1:**
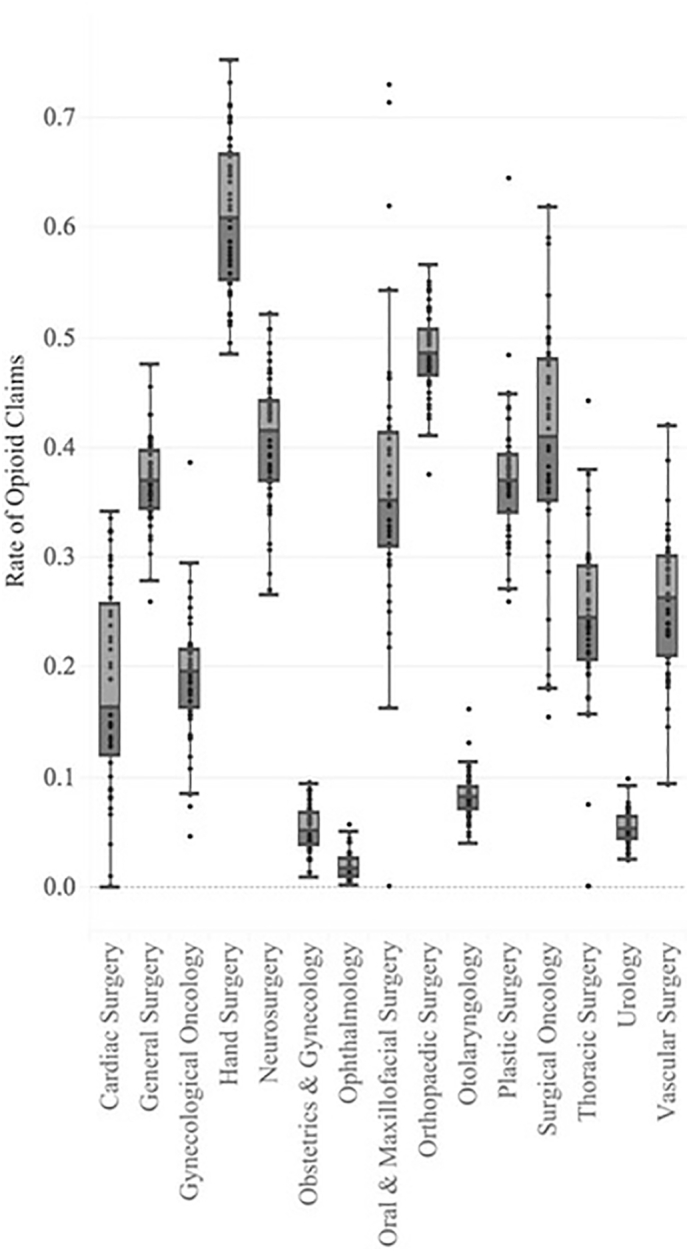
Rate of opioid claims by surgical specialty.

A total of 65,277,932 claims made to Part D by 90,253 surgeons (19,699 obstetrics and gynecology physicians, 14,159 general surgeons, 14,092 orthopedic surgeons, 11,642 ophthalmologists, 7,328 urologists, 7,035 otolaryngologists, 4,246 plastic surgeons, 3,639 neurosurgeons, 2,393 vascular surgeons, 1,806 thoracic surgeons, 1,118 cardiac surgeons, 1,145 hands surgeons, 886 gynecologic oncologists, 713 surgical oncologists, and 352 oral and maxillofacial surgeons) in the 2015 service year were analyzed in this study. Provider subspecialty opioid prescribing practices varied significantly (*P* < .0001), with hand surgeons having a statistically significant higher rate of opioid prescription as compared to all their peers (61.0% of all prescriptions written to Part D beneficiaries, *P* < 0.0001, Fig. 1). Orthopedic surgeons had an opioid prescription rate of 48.6%, significantly greater than all specialties excluding hand surgeons (*P* < .0001). Although significantly less than hand surgeons and orthopedic surgeons, neurosurgeons had an opioid prescription rate of 40.6%, which was significantly greater than all other specialties other than surgical oncology (*P* < .0001). The mean rates of opioid prescription by state are summarized in Fig. 1 by surgical subspecialty. The overall mean rate of opioid prescription for all surgeons in this study was found to be 27.1% (Fig. 1).

### State and Regional Prescribing Practices

A total of 16,738,701 beneficiaries accounted for 65,277,932 claims, a total of 2,201,236,690 days of medication supplies, and $5,659,368,717.00 in total claim costs. During the same year, 5,920,521 claims for 3,362,519 beneficiaries accounted for 66,652,078 of the day supplies of opioid medications and cost Medicare Part D a total of $133,091,997.81 in the calendar year of 2015 on opioid medications (Table 1). Provider state opioid prescribing practices varied significantly (*P* < .05), with Arkansas (36.77%), Colorado (30.63%), Oregon (30.0%), Wisconsin (30.0%), and Idaho (29.06%) having the greatest rates of opioid prescriptions. Maryland (17.69%), Rhode Island (15.23%), New Jersey (13.74%), and New York (13.28%) had significantly lower rates of opioid prescription (*P* < .05). The mean rates of opioid prescription by surgical specialties are summarized in Table 1 by state. City-level prescribing practices have been summarized in Supplemental Table 1 to provide a more detailed understanding of opioid prescribing practices in individual cities.

**Fig. 2:**
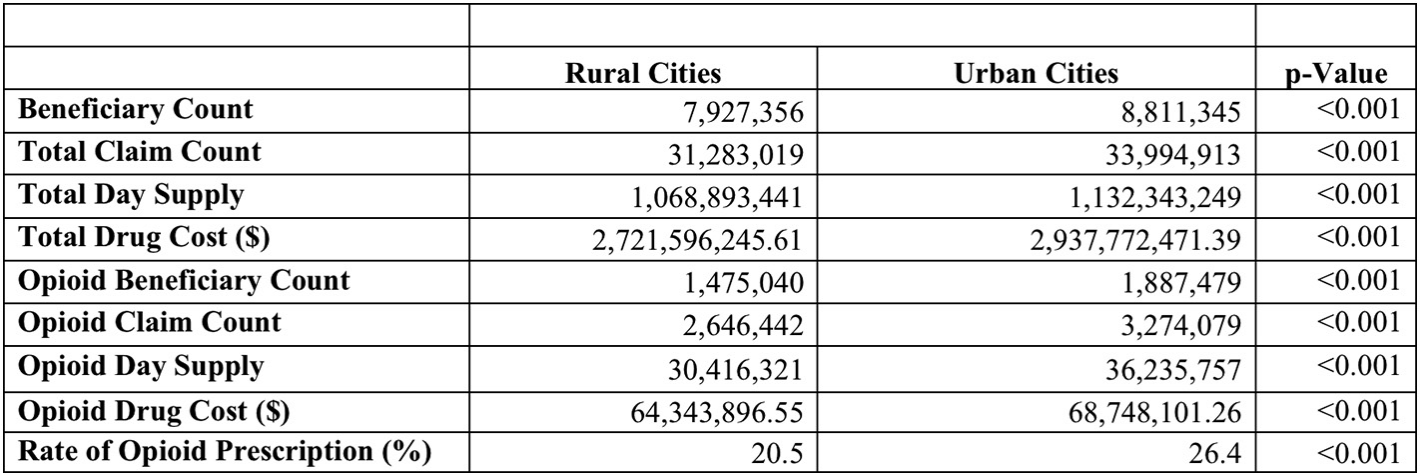
Rates of opioid prescription in rural versus urban practice environments

Furthermore, an analysis of rural versus urban practice environment correlation with rates of opioid prescriptions was calculated; on average, surgeons practicing in urban cities were found to have significantly greater rates of opioid prescription as compared to those practicing in rural areas (Fig. 2). To this end, the rates of opioid prescription by state were compared with the rates of age-adjusted mortality associated with drug overdose, and this relationship demonstrated a statistically significant correlation (*P* < .0001, equation: Opioid Claim Count = 102.882*number + 10,374.7).

**Table 1:** Surgeon opioid prescribing practices by state

*State*	*Beneficiary count*	*Total claim count*	*Total day supply*	*Total drug cost ($)*	*Opioid beneficiary count*	*Opioid claim count*	*Opioid day supply*	*Opioid drug cost ($)*	*Rate of opioid prescription (%)*
AK	9,979	35,676	1,069,782	3,358,628	2,565	4,871	47,319	105,623	36.77
AL	372,787	1,481,754	43,447,933	95,564,758	107,549	221,978	2,993,012	4,330,131	23.07
AR	155,250	615,133	17,157,128	39,117,933	47,179	82,634	820,490	1,438,872	22.52
AZ	301,706	1,054,183	36,913,243	85,926,823	63,063	96,686	937,044	1,825,031	23.49
CA	1,740,127	7,282,374	260,966,145	699,984,725	270,566	455,148	5,376,944	12,067,542	19.99
CO	196,354	691,367	23,076,424	58,383,014	45,502	70,916	640,987	1,453,367	30.63
CT	225,491	873,491	31,874,323	96,019,994	30,472	56,629	669,920	1,439,170	18.33
DC	31,049	135,012	5,071,895	13,682,949	4,481	9,688	150,810	249,028	19.15
DE	53,941	177,058	7,012,611	18,725,125	9,360	14,974	152,792	363,238	19.73
FL	1,463,703	5,492,904	180,517,023	480,701,404	298,604	515,366	6,016,550	14,623,485	21.15
GA	581,701	2,376,499	71,676,808	171,710,803	159,265	294,795	3,344,158	5,700,322	22.95
HI	67,749	271,334	10,735,244	31,422,815	8,664	13,954	130,940	274,994	21.05
IA	144,430	557,647	17,082,392	40,506,094	32,233	51,170	424,902	753,949	23.98
ID	65,973	250,023	8,254,716	15,962,796	18,404	31,827	284,364	566,064	29.06
IL	561,743	2,270,142	79,539,269	198,258,140	99,405	178,153	1,834,368	2,964,229	19.87
IN	329,857	1,214,652	41,470,841	98,054,875	81,795	140,575	1,437,798	2,833,373	25.76
KS	133,861	535,981	16,816,076	37,359,917	30,952	53,515	511,197	1,115,005	19.78
KY	263,590	1,001,200	30,693,795	76,352,075	71,990	125,448	1,437,834	2,685,767	19.45
LA	342,587	1,591,354	47,826,099	107,083,023	81,765	193,741	3,032,296	4,178,334	18.81
MA	353,228	1,443,568	50,525,510	118,824,799	47,494	80,054	805,895	1,324,997	21.20
MD	261,845	928,476	35,620,086	99,509,535	39,597	71,136	849,093	2,463,334	17.69
ME	57,917	194,847	7,057,665	19,510,658	11,965	18,832	159,303	310,826	27.62
MI	599,880	2,121,024	76,555,908	174,665,701	134,021	237,988	2,963,930	5,469,915	23.31
MN	232,288	819,268	26,726,316	59,934,776	51,384	74,330	544,824	1,118,266	26.54
MO	367,005	1,434,046	45,414,624	109,780,180	99,952	176,817	1,855,696	4,575,567	26.27
MS	180,069	747,010	21,510,023	43,203,292	45,242	78,985	800,621	1,205,327	20.17
MT	41,047	150,138	4,767,981	9,838,930	10,212	16,027	126,448	254,024	21.88
NC	574,505	2,205,820	69,001,595	173,185,149	129,947	231,635	2,882,539	5,518,812	21.88
ND	31,244	122,071	3,699,895	7,969,964	6,201	9,923	82,198	146,032	22.71
NE	86,376	336,715	10,104,050	24,843,741	17,565	27,383	212,976	414,863	24.43
NH	63,054	230,143	7,957,996	19,017,392	10,472	17,453	147,823	256,073	22.99
NJ	507,572	1,921,748	73,758,380	231,260,881	67,564	116,554	1,237,555	3,406,075	13.74
NM	73,990	287,462	9,744,584	20,563,532	13,920	21,549	206,002	315,147	23.54
NV	105,944	388,673	13,350,559	30,236,836	21,055	32,793	350,120	682,541	22.22
NY	1,203,243	5,012,336	181,973,169	552,636,405	114,286	176,740	2,076,484	4,621,963	13.28
OH	681,452	2,557,174	90,348,272	201,037,916	143,388	262,016	3,025,418	6,652,703	22.41
OK	180,135	702,912	22,557,474	57,986,471	54,376	109,232	1,305,326	3,154,103	22.82
OR	195,971	724,582	24,811,151	51,056,798	42,120	65,230	509,286	1,059,254	30.00
PA	830,064	3,184,103	108,409,029	292,038,149	147,853	267,937	3,141,709	8,069,401	17.99
RI	64,628	267,863	8,161,707	17,500,835	10,518	19,065	205,326	377,717	15.23
SC	322,119	1,244,145	38,698,707	103,567,252	85,997	153,999	1,798,714	3,501,407	22.80
SD	40,330	146,325	4,467,453	8,997,141	8,206	12,776	101,492	167,547	23.57
TN	425,243	1,610,179	50,510,566	122,971,881	108,542	186,414	1,970,966	3,819,546	22.97
TX	1,137,739	4,514,799	148,917,551	422,798,942	244,690	451,192	5,184,077	7,950,131	22.53
UT	86,612	303,008	9,552,959	20,348,430	21,730	36,091	331,000	674,208	23.82
VA	354,098	1,354,985	46,695,583	111,324,376	71,032	122,351	1,280,326	2,261,115	21.16
VT	27,293	107,060	3,888,569	9,680,490	3,047	4,999	39,172	72,505	20.89
WA	234,379	854,035	28,109,658	65,749,947	55,465	85,868	676,714	1,338,408	24.64
WI	247,464	925,882	30,394,981	70,366,349	50,173	85,007	794,714	1,616,850	30.00
WV	112,875	464,509	14,512,559	35,840,797	26,222	49,794	655,064	1,167,862	20.89
WY	17,214	65,242	2,230,383	4,945,280	4,469	8,283	87,542	157,952	25.77

## DISCUSSION

The 65,277,932 claims made to Part D by 90,253 surgeons in the 2015 service year were analyzed in this study, demonstrating statistically significant differences in the opioid prescription prescribing practices of surgeons from different states, cities, practice settings, and subspecialties (*P* < .05). In 2015, these surgeons' opioid medication claims cost the health care system $133,091,997.81 in drug benefits. Based upon our analysis of the CDC's drug-related mortality data, these prescriptions may have cost the system much more.

The importance of high opioid prescription rates by surgeons cannot be emphasized enough because, although opiates can be the most effective pain management option in the short term, opiates present serious risks with initiation and continued use. These risks are the well-documented and national epidemic of dependence and overdose-related mortality [10]. In the year of 2013 alone, an estimated 1.9 million people were dependent on or abused prescription opiates. Between 1999 and 2014, more than 165,000 people died from overdose deaths related to opioid pain medications [1], [11], [12]. Of all causes of accidental deaths, drug overdose has become the leading cause with 52,404 lethal drug overdoses in 2015 alone. Of these lethal drug overdose deaths, opioid overdose is the single greatest contributor, accounting for 20,101 deaths, or 38% in total [2]. In 2010, a Substance Abuse and Mental Health Services Administration survey reported that at least 16 million Americans had taken a prescription pain medication or non–medically indicated opioid at least 1 time in the past year, with 55% of these people also indicating that they procured the medication from a friend or relative free of cost. Furthermore, 17.3% of these people reported that they were given prescriptions for these pain reliever medications from a doctor [13]. In 2010, the *New England Journal of Medicine* reported a study that of the 2.4 million opioid-dependent people in the United States, 60% of their opioids were obtained either directly or indirectly from a physician prescription [14].

With regards to the distinct disparities in prescriptive tendencies between surgeon types, addressing the statistically significant rates of hand, orthopedic, and neurosurgeons could be helpful to conjecture that there is a need for improved pain management options for musculoskeletal diagnoses. Given the widespread knowledge that musculoskeletal diagnoses are commonly associated with opioid prescriptions and repeat opioid prescriptions, there is significant room for improvement of musculoskeletal pain management. Furthermore, Khalid et al found that there were no statistically significant regional differences between neurosurgeons prescribing opiates, which is interesting given the stark differences between the subspecialties most likely to prescribe opiates [16].

Of note and surprise, among the Part D beneficiary population, there are high rates of opioid prescription in urban cities at 26.4% compared to rural cities with 20.5% (*P* < .001) (Fig. 2). Anecdotally, there has been more focus on rural opioid addiction and overdose, which could indicate either a change as the opioid epidemic matures or a difference in opiate use in the general population compared to the CMS population.

The opioid epidemic is a complex issue affecting our country, with serious implications for public health and safety [1], [15]. There are already federal, state, and institutional initiatives directed at developing appropriate responses to this crisis. For surgeons, the vast majority of our patients present with pain or pain-associated syndromes, and accordingly, most of our interventions require some course of opioid medications. As such, being aware of our prescribing practices is the first step to develop strategies to address this epidemic. The sparse number of surgical studies demonstrating optimization of opioid prescription practices is also an area of study that could use greater attention and resources with high-level clinical studies to direct outcomes-based practice. Different surgical specialties should be aware of their rates of prescription as they compare to their peers. Likewise, different practice environments, cities, and states, although are treating many of the same conditions and providing a high standard of care, have a different approach to opioid prescription. It is our hope that the knowledge of the rates on a national context will help introspective adjustment and influence regulation and policy to help mitigate the rise in opioid-related deaths.

### Study Limitations

The Part D Prescriber PUF detail file does not include claim, medication cost, or day supply associated with fewer than 11 Part D drug claims per provider; thus, the data presented here unfortunately underestimate the true Part D totals. Furthermore, most Medicare Part D beneficiaries are older (> 65 years old), so these patients may represent more significant or at least advanced chronic pathology, with longer recovery times requiring more pain control than the general populace.

In conclusion, those with opioid prescribing privileges are given great responsibility, with the responsibility and authority to use these medications in safe, efficacious, and prudent manner in the treatment of their patients; therefore, physicians have an important role to play in responding to the opioid crisis. Surgeons have the opportunity to help to ensure safe and effective use and deter its abuse, especially in the acute context of their practice and among the elderly CMS population that is susceptible to opioid overdose. Further research in the opioid prescribing habits of surgical subspecialties in the general population outside of Part D plans would be highly beneficial for future studies to pursue.

The following are the supplementary data related to this article.

Supplemental Table 1 City-level prescribing practices of all surgeon providers Supplemental Table 1

## Author Contribution

• Syed Ibad Khalid, MD: conceived and designed the analysis, collected the data, contributed data or analysis tools, performed the analysis, wrote the paper, approved the final version of the paper
• Ryan Kelly, BS: collected the data, contributed data or analysis tools, performed the analysis
• Rita Wu, BS and Amelia Y. Ni, BA BS: collected the data, wrote the paper
• Ridha Khalid, MD: conceived and designed the analysis, wrote the paper, approved the final version of the paper
• Owoicho Adogwa, MD, MPH: conceived and designed the analysis, wrote the paper, approved the final version of the paper
• Joseph S. Cheng, MD, MS: conceived and designed the analysis, approved the final version of the paper

## Conflict of Interest

• No authors have any conflicts of interest to report.

## Funding Sources

• No external funding was used to support this study.

## References

[1] Dowell D., Haegerich T.M., Chou R. (2016). CDC guideline for prescribing opioids for chronic pain—United States, 2016. JAMA.

[2] Rudd R.A., Aleshire N., Zibbell J.E., Increases in Drug Gladden R.M. (2016). Opioid overdose deaths—United States, 2000–2014. MMWR Morb Mortal Wkly Rep.

[5] Paulozzi L.J., Jones C.M., Mack K.A., Rudd R.A. (2011). Vital signs: overdoses of prescription opioid pain relievers—United States, 1999–2008. MMWR Morb Mortal Wkly Rep.

[6] Daubresse M., Chang H.-Y., Yu Y., Viswanathan S., Shah N.D., Stafford R.S. (2013). Ambulatory diagnosis and treatment of nonmalignant pain in the United States, 2000–2010. Med Care.

[7] Gaskell H., Derry S., Moore R.A., McQuay H.J. (2009). Single dose oral oxycodone and oxycodone plus paracetamol (acetaminophen) for acute postoperative pain in adults. Cochrane Database Syst Rev.

[8] Korn S., Vassil T.C., Kotey P.N., Fricke J.R. (2004). Comparison of rofecoxib and oxycodone plus acetaminophen in the treatment of acute pain: a randomized, double-blind, placebo-controlled study in patients with moderate to severe postoperative pain in the third molar extraction model. Clin Ther.

[9] Moore RA, Derry S, Aldington D, Wiffen PJ. Single dose oral analgesics for acute postoperative pain in adults—an overview of Cochrane reviews. Cochrane Database Syst Rev 2015(9). 10.1002/14651858.CD008659.pub3 PMC6485441 26414123

[10] Murthy VH. Surgeon General's report on alcohol, drugs, and health (2017). JAMA.

[11] Herzberg D., Guarino H., Mateu-Gelabert P., Bennett A.S. (2016). Recurring epidemics of pharmaceutical drug abuse in America: time for an all-drug strategy. Am J Public Health.

[12] Seligman J., Felder S.S., Robinson M.E. (2015). Substance Abuse and Mental Health Services Administration (SAMHSA) behavioral health disaster response app. Disaster Med Public Health Prep.

[13] Aldworth J., Colpe L.J., Gfroerer J.C., Novak S.P., Chromy J.R., Barker P.R. (2010). The National survey on drug use and health mental health surveillance study: calibration analysis. Int J Methods Psychiatr Res.

[14] Ballantyne JC, Sullivan MD. Why doctors prescribe opioids to known opioid abusers. *N Engl J Med* 368(5):484–485, 2013. 10.1056/NEJMc1214553 23363517

[15] Jones C.M., Campopiano M., Baldwin G., McCance-Katz E. (2015). National and state treatment need and capacity for opioid agonist medication-assisted treatment. Am J Public Health.

[16] Khalid S.I., Adogwa O., Elsamadicy A.A., Mehta A.I., Cheng J. (2018). Opioid prescribing practices of neurosurgeons: an analysis of Medicare Part D. World Neurosurg.

